# Colour vision and background adaptation in a passerine bird, the zebra finch (*Taeniopygia guttata*)

**DOI:** 10.1098/rsos.160383

**Published:** 2016-09-14

**Authors:** Olle Lind

**Affiliations:** Department of Philosophy, Cognitive science, Lund University, Lund, Sweden

**Keywords:** colour vision, threshold, background adaptation, behavioural test, passerine bird

## Abstract

Today, there is good knowledge of the physiological basis of bird colour vision and how mathematical models can be used to predict visual thresholds. However, we still know only little about how colour vision changes between different viewing conditions. This limits the understanding of how colour signalling is configured in habitats where the light of the illumination and the background may shift dramatically. I examined how colour discrimination in zebra finch (*Taeniopygia guttata*) is affected by adaptation to different backgrounds. I trained finches in a two-alternative choice task, to choose between red discs displayed on backgrounds with different colours. I found that discrimination thresholds correlate with stimulus contrast to the background. Thresholds are low, and in agreement with model predictions, for a background with a red colour similar to the discs. For the most contrasting green background, thresholds are about five times higher than this. Subsequently, I trained the finches for the detection of single discs on a grey background. Detection thresholds are about 2.5 to 3 times higher than discrimination thresholds. This study demonstrates close similarities in human and bird colour vision, and the quantitative data offer a new possibility to account for shifting viewing conditions in colour vision models.

## 1. Introduction

Many animals use colourful ornaments as signals to attract the opposite sex during mate choice. How such colour traits emerge and how trait variation is maintained are core questions for evolutionary biology [1,2]. Birds are a central taxon in this context, with a large number of studies of numerous aspects of feather coloration [3]. Unfortunately, plumage colours cannot be fully appreciated by human perception as we have very different colour vision compared with birds [4]. Instead, we represent the bird view of colours by mathematical models, developed from an understanding of the physiological basis of colour vision.

The bird retina has four spectrally distinct cone photoreceptors that mediate tetrachromatic colour vision; the UVS/VS (ultraviolet or violet-sensitive), the SWS (short wavelength-sensitive), the MWS (medium wavelength-sensitive) and the LWS (long wavelength-sensitive) cone [5]. There are also double cones and rods that are used for brightness and spatial vision (but not colour vision) in bright and dim light, respectively. Birds not only have more cone types than trichromatic humans, the cones also differ in morphology in birds and are equipped with carotenoid-pigmented oil droplets (the oil droplets lack pigmentation in UVS/VS cones [5]) at the distal end of the inner segments. The oil droplets filter incident light before it reaches the visual pigments and alter cone sensitivity, which is believed to improve spectral resolution and colour constancy at the cost of a lower absolute sensitivity [6–8].

Careful descriptions of accurate cone sensitivities are fundamental to colour vision models. Another requirement is the validation of model predictions by behavioural data on visual performance. Today, the most popular model of animal colour vision is the receptor noise-limited (RNL) model proposed by Vorobyev & Osorio [9]. This model agrees well with behavioural data on spectral sensitivity in red-billed leiothrix and budgerigars [9–11], as well as the discrimination of object colours in domestic chicks [12]. However, to explore colour vision in a natural context, such as the perception of colour traits displayed in a habitat of variable vegetation, the models need to account for the effect of photoreceptor adaptation to the visual background. To this date, this has been a considerable limitation of the RNL model.

From studies on human vision, it is clear that colour discrimination changes when the photoreceptors are adapted to different backgrounds. Thresholds are low when discrimination is made between stimuli with colours similar to the adaptive background, and higher if the stimuli and the background are more different [13,14]. Described as such, the stimuli and the background are separate variables and this effect should not be confused with Weber's law that postulates a constant ratio between just notable stimulus change and stimulus intensity. The change in discrimination over backgrounds could have far-reaching consequences for how animal colour vision and colour signalling are configured and understood. Coloured feathers are often strongly contrasting the background, which makes them conspicuous. Similarly, fruits are usually clearly distinct from the surrounding vegetation when ripe [15]. How well can birds discern the variation among such traits?

In this study, I used behavioural experiments to test how colour discrimination relates to background adaptation in zebra finches (*Taeniopygia guttata*). Male zebra finches have red beaks that are used as a sexual signal during mate choice [16]. Females prefer males that have more red beaks, but also red leg rings [17,18]. I exploited this seemingly general colour preference by training female zebra finches to discriminate between red colour discs of different saturation displayed on backgrounds with different colour and brightness.

## 2. Material and methods

### 2.1. Animals

I used six adult female zebra finches identified as b12, b35, w51, w60, y77 and y82. The experiments were carried out at Lund University field station (Stensoffa) between February and April 2016. I trained and tested birds during the weekdays, and on these days birds were only fed in the experiments except for a small portion of millet seeds that was given in the evening. Food was available ad libitum during weekends, and water was freely accessible. I kept birds in pairs in adjacent cages under a 14 L : 10 D light regime with daylight at approximately 400 lux.

### 2.2. Apparatus

**Figure 1.**
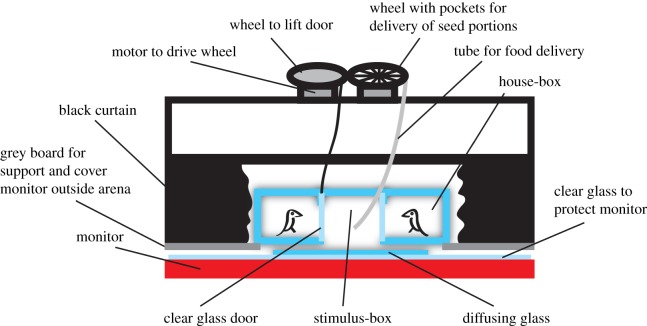
The apparatus as viewed from the side with the curtains ‘withdrawn’ to show the arena. The arena rested on grey boards about 20 mm above a diffusing glass floor on the monitor. An extra layer of clear glass protected the monitor from mechanical damage while the diffusing glass could be removed for cleaning. Two tubes (only one shown) were used to deliver seeds from the wheel to the left or the right side of the stimulus-box. Transparent doors closed the house-boxes, but could be opened by an attachment (only one shown) to a wheel similar to that used for feeding. See electronic supplementary material, methods for photographs (electronic supplementary material, figure S1). The arena was solely illuminated by the monitor.

I tested birds in a plastic-glass arena (figure 1) that was built from 3 mm thick diffusing glass (polymethyl acrylate, Biltema, Lund, Sweden) and 2 mm clear glass (Styrene Acrylonitrile, Biltema). Birds waited inside ‘house-boxes’ (150 mm width, 150 mm length, 100 mm height) made out of diffusing glass at the bottom, back and sides, and clear glass at the top and the front that faced the ‘stimulus-box’ (figure 1). Birds accessed the stimulus-box through openings in the front glass that could be shut by clear glass doors, which were slid vertically using DC motors (Dynamixel AX-12, Robotis, Seoul, South Korea). The stimulus-box (180 mm width, 150 mm length, 120 mm height) was made out of diffusing glass on the sides and clear glass at the top. The floor of the stimulus-box was diffusing glass (300 mm width, 600 mm length) placed on a horizontally placed monitor (32WL30MS, LG, Seoul, South Korea). The outer parts of the housing-boxes rested on grey board so that the whole arena was held 20 mm above the diffusing glass floor. This arrangement allowed the diffusing glass to be cleaned from seeds regularly by blowing in air from the side. Between sessions, I could remove and rinse both the arena and the diffusing glass floor.

Grey wood boards covered the monitor at the sides of the arena, and the whole arena was flanked by black curtains, so that all light entering the boxes came from directly below (figure 1). The apparatus was placed in a dark room with the computer screen held at the dimmest level (radiance from the stimulus background with the monitor off was less than 0.01 cd m^−2^).

Two feeder tubes connected to feeder wheels entered the stimulus-box at each side. Following correct answers, a DC motor (same as above) turned the feeder-wheel so that 3–6 millet seeds entered the feeder tube and dropped on the stimulus-box floor close to the rewarded stimulus. I controlled the screen and the motors by custom-written software written in Matlab (R2014b, MathWorks, Natick, MA, USA). All scripts are available on request.

### 2.3. Stimuli

Stimuli were uniformly coloured discs with a diameter of 40 mm, and a centre-to-centre distance of 85 mm, displayed on the monitor at the middle of the stimulus-box (figure 1). I used large discs (visual angle roughly 11° at 200 mm distance) to ensure high chromatic contrast sensitivity [19]. The edges of the discs were made diffuse by the transmission through the diffusing glass floor (figure 1). Discs were visible for 5 s after a correct choice, or disappeared immediately after incorrect choices. I measured stimulus and background radiance (µW cm^−2^ nm^−1^ sr^−1^) with a spectroradiometer (ILT950, International Light Technologies, Peabody, MA, USA) at several angles, at both sides of the stimulus-box, and both at the beginning and at the end of the experiments. I found only negligible variation between these measurements, and used an average for both sides, taken at an angle of approximately 60° at the end of the experiments for further analysis. Moreover, I measured radiance without the removable windows, to represent the view of the birds just prior to choosing. However, colour contrast changes by less than 1% when the windows are inserted.

### 2.4. Modelling colour contrast

I used the RNL model to calculate colour contrast [9]. Below are the equations used for the calculations, while a detailed model description can be found elsewhere [4,9].

The sensitivity, *R*, of a photoreceptor is

2.1 $$R_{i}(\lambda )=r_{i}(\lambda )p_{i}(\lambda )o(\lambda ),$$

where *r* is the normalized absorbance spectrum of the visual pigment in receptor *i* (*i* = UVS, SWS, MWS, LWS), *p* is oil droplet transmittance, *o* is ocular media transmittance and λ is wavelength in nanometres. Visual pigment sensitivity was estimated using the Govardovskii template [20], and oil droplets were modelled as cut-off filters [21], together with data from microspectrophotometry [22]. Data on ocular media transmittance were taken from [23]. I assume that self-screening of the visual pigment is negligible [24]. Data on cone sensitivities are given in the electronic supplementary material.

The quantum catch of a cone, *Q_*i*_*, for a visual stimulus, *L*, is

2.2 $$Q_{i}=k_{i}\int_{300}^{700}R_{i}(\lambda )L(\lambda )\;d\lambda ,$$

where *L* is defined in the unit of quantum radiance (quanta cm^−2^ nm^−1^ sr^−1^) and *k* is a scaling coefficient for receptor adaptation to the background, *L*_b_

2.3 $$k_{i}=\frac{1}{\int_{300}^{700}R_{i}(\lambda )L_{b}(\lambda )\;d\lambda }.$$

To plot colours in a chromaticity diagram (for details, see [4]), intensity differences are disregarded and only relative quantum catches are used

2.4 $$q_{i}=\frac{Q_{i}}{Q_{\mathrm{UVS}}+Q_{\mathrm{SWS}}+Q_{\mathrm{MWS}}+Q_{\mathrm{LWS}}}.$$

To calculate chromatic contrast between stimuli or between stimulus and background, the receptor-specific contrast Δ*f*, is determined

2.5 $$\Delta f_{i}=\ln\left(\frac{Q_{i,L1}}{Q_{i,L2}}\right).$$

In the RNL model, colour discrimination is constrained by receptor noise, which can be considered as the limiting Weber fraction, *ω,* in each receptor mechanism

2.6 $$\omega_{i}=\frac{v_{i}}{\sqrt{\eta_{i}}}\;,$$

where *v* is the noise-to-signal ratio of an individual cone and *η* is the number of this cone type per receptive field (cone abundance). I used a Weber fraction of 0.1 in the LWS mechanism, which is the noise estimate for red-billed leiothrix and budgerigar in earlier studies of spectral sensitivity thresholds [11,25] a cone abundance ratio of 1 : 1.5 : 2 : 3 [22]. The colour discrimination threshold, Δ*S^*t*^,* is calculated as [9]

2.7 $$\begin{array}{rl} {\left(\Delta S^{t}\right)}^{2} & ={\left(\omega_{\mathrm{UVS}}\omega_{\mathrm{SWS}}\right)}^{2}{\left(\Delta f_{\mathrm{LWS}}-\Delta f_{\mathrm{MWS}}\right)}^{2}+{\left(\omega_{\mathrm{UVS}}\omega_{\mathrm{MWS}}\right)}^{2}{\left(\Delta f_{\mathrm{LWS}}-\Delta f_{\mathrm{SWS}}\right)}^{2}\\ & \;+{\left(\omega_{\mathrm{UVS}}\omega_{\mathrm{LWS}}\right)}^{2}{\left(\Delta f_{\mathrm{MWS}}-\Delta f_{\mathrm{SWS}}\right)}^{2}+{\left(\omega_{\mathrm{SWS}}\omega_{\mathrm{MWS}}\right)}^{2}{\left(\Delta f_{\mathrm{LWS}}-\Delta f_{\mathrm{UVS}}\right)}^{2}\\ & \;+\frac{{\left(\omega_{\mathrm{SWS}}\omega_{\mathrm{LWS}}\right)}^{2}{\left(\Delta f_{\mathrm{MWS}}-\Delta f_{\mathrm{UVS}}\right)}^{2}+{\left(\omega_{\mathrm{MWS}}\omega_{\mathrm{LWS}}\right)}^{2}{\left(\Delta f_{\mathrm{SWS}}-\Delta f_{\mathrm{UVS}}\right)}^{2}}{{\left(\omega_{\mathrm{UVS}}\omega_{\mathrm{SWS}}\omega_{\mathrm{MWS}}\right)}^{2}+{\left(\omega_{\mathrm{UVS}}\omega_{\mathrm{SWS}}\omega_{\mathrm{LWS}}\right)}^{2}+{\left(\omega_{\mathrm{UVS}}\omega_{\mathrm{MWS}}\omega_{\mathrm{LWS}}\right)}^{2}+{\left(\omega_{\mathrm{SWS}}\omega_{\mathrm{MWS}}\omega_{\mathrm{LWS}}\right)}^{2}}. \end{array}$$

Colour contrast is given in the unit of JND (just notable difference), where 1 JND is defined as the discrimination threshold.

### 2.5. Procedure

Birds were trained and tested in pairs. Each pair was adapted to the experimental conditions for 5 min in the house-boxes before training or tests commenced. At the very first arena encounters, birds were freely exploring the boxes, with millet seeds placed on the rewarded stimulus in the stimulus-box. Next, birds were trained to enter the stimulus-box separately, eat from the millet seeds at the rewarded stimulus, and return to the house-box to initiate new trials. In subsequent training, birds were required to choose the rewarded stimulus to receive the food reward, and the choices were recorded. Birds were tested for five trials before the other bird in the pair was tested under the same conditions, and all trials could be observed by the waiting bird. Sessions comprised between 10 and 50 trials and were ended when any of the birds ceased to choose stimuli during presentations, or displayed signs of fatigue such as side bias or stereotypic behaviour. In total, I ran two to four sessions each day and rearranged pairs so that birds at the same level of progress were matched to advance training efficiently.

Trials started with a three-tick auditory signal together with the presentation of the stimuli. After 3 s, I opened the transparent door and the bird entered the stimulus-box and made a choice by turning towards either of the discs. The stimuli were hidden to me as I was operating the computer behind the black curtain (figure 1), but I could see the upper parts of the arena and track the movement of the bird. I entered the choice (left or right side) into Matlab, where pre-set scripts recorded the data, determined if the correct stimulus was chosen (this was blind to me) and if so, immediately delivered a food reward. For incorrect choices, a low frequency sound was played and the stimuli were removed. In both cases, the bird returned into the house-box to initiate a new trial.

For the discrimination series 1, I first trained birds to choose between a rewarded red disc and a non-rewarded grey disc (contrast, 6.9 JNDs) on a grey background. The criterion for completing this training was at least 80% correct choices during 40 consecutive trials. At training level 2, birds were trained with the red disc, but now the grey disc was replaced by a non-rewarded red-tinted disc (contrast, 3.7 JNDs) on the same grey background. By the same criterion, birds were advanced to train on backgrounds with yellow and green colour. On these backgrounds, I showed the bird the same rewarded red disc together with either the grey, or the red-tinted discs, both occurring randomly but at the same average frequency. For this generalization training, and for all subsequent training, birds completed training by a less stringent criterion of at least 80% correct choices during 20 consecutive trials.

**Figure 2.**
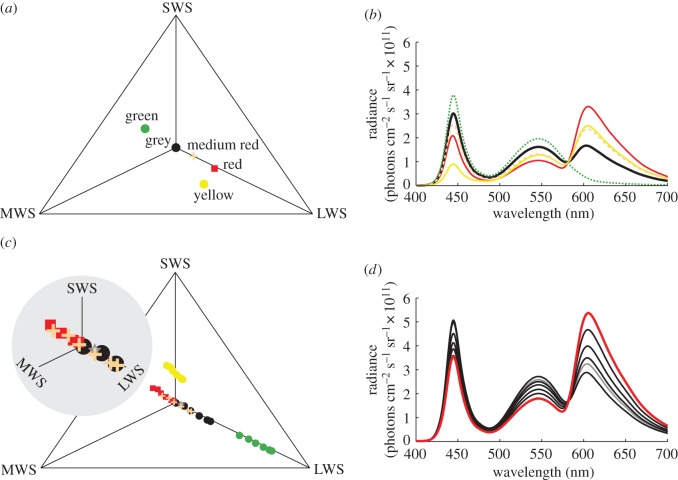
Chromaticity diagrams and the corresponding colour spectra of the backgrounds (*a,b*), and the discs (*c,d*). The colour loci depend on their relative excitation of the SWS, MWS and LWS cones. The quantum catch of the UVS cone is negligible and not shown. The relative positions of the background colours are shown in (*a*) with the receptors adapted (equation (2.3)) to the grey background (neutral point at the diagram centre). Background spectra (*b*) are; red (red line), medium red (dashed light-red line), yellow (yellow line), grey (thick black line) and green background (green dotted line). Colour loci of discs (*c*) are colour coded according to which background they appear on. In panel (*c*), receptor adaptation brings the background colour to the neutral point so that the relative positions of the disc colour loci shift. The inset in panel (*c*) shows a magnified view of the crowded region at the centre of the diagram. The set of disc colour spectra (*d*) were the same for tests on all backgrounds, except for a small ‘red tint’ added to the unsaturated disc used in the detection task (grey star in (*c*) and grey line in (*d*)). In panel (*d*), the saturation of the red colours of the discs correlates with decreasing amplitude in the spectra at 550 nm, and the thick red line indicates the reinforced disc colour. Data on colour spectra are given in the electronic supplementary material.

When all training was completed, I tested the birds on the grey, yellow, green and a brighter variant of the grey background. The rewarded stimulus was always the highly saturated red disc, while the non-rewarded alternative was a disc chosen at random from a series of differently saturated discs ranging from grey to red at contrasts of 0.1, 0.6, 2.1, 3.7, 5.2 and 6.9 JNDs (figure 2). All stimuli were balanced for approximately equal intensity for double cones, with Michelson contrasts of less than 4%. Michelson contrast, *C*, is defined as

2.8 $$C=\frac{Q_{D,L1}-Q_{D,L2}}{Q_{D,L1}+Q_{D,L2}},$$

where *Q*_D_ is quantum catch of double cones and *L*1 is the stimulus with the highest intensity.

The rewarded disc was brighter than the background with a Michelson contrast of 29%, 35%, 29% and 16% for the grey, yellow, green and bright grey backgrounds, respectively.

In tests, bird pairs were assigned one of the backgrounds randomly each day, and no background was tested over two consecutive days (except when all tests at other backgrounds were completed). At the beginning of each session, each bird received five ‘instruction’ trials at the strongest contrast (6.9 JNDs) that were not recorded.

The position of the rewarded stimulus (left or right) was determined following a random assembly of Fellows series [26] while avoiding more than four rewarded repetitions at any side. Schedules were pre-programmed in Matlab and executed blind to me during the experiments. Tests were completed when birds had made 30 choices for each contrast at each of the four backgrounds.

In discrimination series 2, birds were first trained to discriminate between the discs displayed on a saturated red background with the same chromaticity (same locus in the chromaticity diagram, figure 2), but dimmer (Michelson contrast of 25%) than the rewarded disc (figure 2). As this training failed (see Results), I retrained the birds on a dim (Michelson contrast of 28%) ‘medium red’ background that was less saturated than the rewarded disc (figure 2).

In the detection series, birds were trained to detect a single disc with a contrast of either 6.8 or 4.8 JNDs against a background with the colour used for the completely unsaturated grey colour stimulus in the previous experiments. In tests, the rewarded stimuli were all the non-rewarded stimuli used in the earlier tests, except for a slight LWS-cone contrast added to the most unsaturated colour (figure 2*a,c*). The contrasts between this saturation series and the grey background were 1.0, 1.7, 3.1, 4.8, 6.3 and 6.8 JNDs.

After the completion of test series 3, birds were immediately tested, without training, for the detection of grey discs that were dimmer than the grey background at Michelson contrasts of 1.7%, 5.4%, 12%, 23%, 40% and 59%.

### 2.6. Psychometric analysis

I assume that the data follow a binomial distribution and set the threshold to 66.7% correct choices (one-tailed binomial distribution, *n* = 30, *p* < 0.05). This threshold was interpolated from a logistic psychometric function fitted to the data

2.9 $$\psi (x)=\gamma +(1-\gamma -\lambda )\frac{1}{1+e^{a-x/b}}\;,$$

where *ψ* is the correct choice frequency at stimulus intensity *x*, *γ* is the guess rate, the lower asymptote of the function (fixed to 0.5), λ is the lapse rate, the difference between the upper asymptote and 1 (allowed to vary between 0 and 0.25), and *a* and *b* are unrestricted parameters representing slope position and steepness, respectively. The psychometric function was fitted to the behavioural data using a maximal-likelihood procedure executed by a modified version of the free Matlab program Palamedes (v. 1.5.0 [27]).

## 3. Results

### 3.1. Discrimination series 1

All birds successfully passed training on the grey, yellow and green backgrounds, except one bird that failed to complete training level 2 on the grey background (electronic supplementary material, S2). I decided to use this bird as company for other birds in the tests, thus carrying out all procedures but ignoring the results.

**Figure 3.**
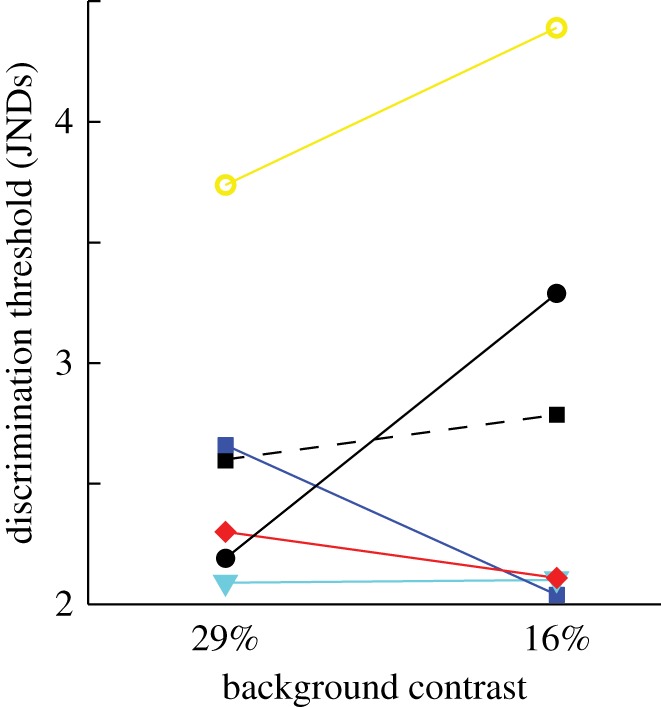
The discrimination thresholds for five individual zebra finches (w60, yellow circles; y77, blue squares; w51, red diamonds; y82, black circles and b35, cyan triangles) on a grey and a bright grey background with Michelson contrast of 29% and 16% against the rewarded stimulus. There is no difference in average threshold (black squares) between the conditions (Wilcoxon, rank sum = 29, *p* = 0.84). Log_10_ quantum catch of double cones for the backgrounds are 13.15 and 13.28 (32.5 cd m^−2^ and 42.9 cd m^−2^).

I tested birds on grey, bright grey, yellow and green backgrounds. First, I compared the thresholds obtained for the grey backgrounds of different intensity. This shift in achromatic contrast did not cause any change of discrimination thresholds (figure 3).

**Figure 4.**
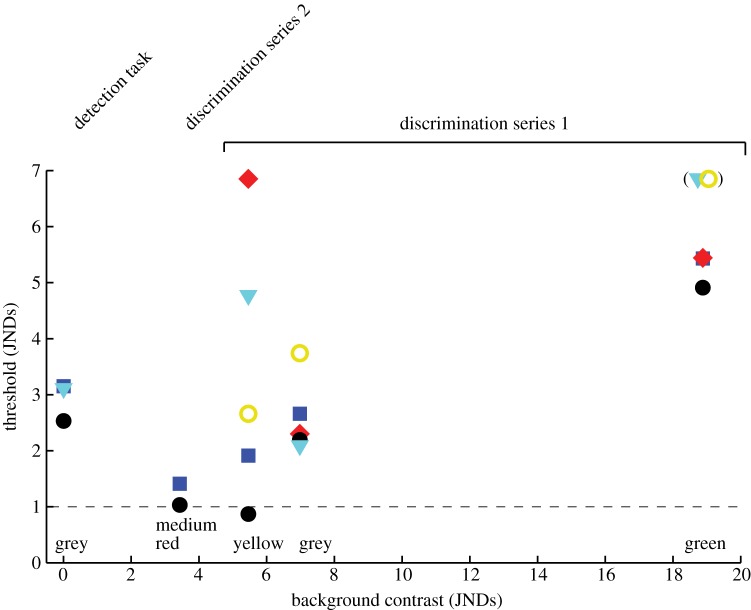
Colour difference thresholds as a function of the contrast between the background and the rewarded stimulus. Each symbol represents the detection (left) or the discrimination (right) threshold of an individual zebra finch (colour coded as in figure 3). Please note the coinciding symbols for the green background. Colour names at the *x*-axis refer to the background colour. The parenthesis indicates birds that failed to discriminate any contrast at the green background. Log_10_ quantum catch of double cones for the backgrounds are: grey (detection task), 13.38; medium red, 13.16; yellow, 13.16; grey, 13.15; green, 13.09 (54.9 cd m^−2^, 31.7 cd m^−2^, 31 cd m^−2^, 32.5 cd m^−2^, 30.7 cd m^−2^).

Despite being successful in training (electronic supplementary material, S2), two birds (w60 and b35) failed to produce any correct choice frequencies significantly above random level in the tests on the green background (figure 4). For the yellow background, one bird (w51) performed poorly (electronic supplementary material, S5), and a meaningful psychometric function could not be fitted to the data. Instead, the threshold was set to the highest contrast of 6.9 JNDs, which was the only level that gave significant choice behaviour for this bird for this particular background.

The thresholds for discrimination series 1 correlate positively with background contrast in three birds, y82, y77 and w60. Thus, thresholds are lowest for the yellow, intermediate for the grey, and highest for the green background (figure 4). For the other two birds, w51 and b35, thresholds are lowest on the grey background, and higher on both the yellow and the green backgrounds (figure 4).

### 3.2. Discrimination series 2

I trained birds to discriminate the same set of stimuli as previously, but now on a red background that was less radiant but of the same chromaticity as the rewarded stimulus. All birds were confused by these conditions, and training was aborted after several hundreds of trials with no positive learning trend (electronic supplementary material, S3).

Instead, I trained the birds to carry out the discrimination task on a less saturated ‘medium red’ background (figure 2) and now, the two best-performing birds (y77 and y82) quickly completed training (electronic supplementary material, S4). I decided to test y77 and y82 for this background and advance the other birds to the task of chromatic detection (see below). The discrimination thresholds for the medium red background are close to the theoretical limit of 1 JND set by the RNL model (figure 4).

### 3.3. Chromatic detection

**Figure 5.**
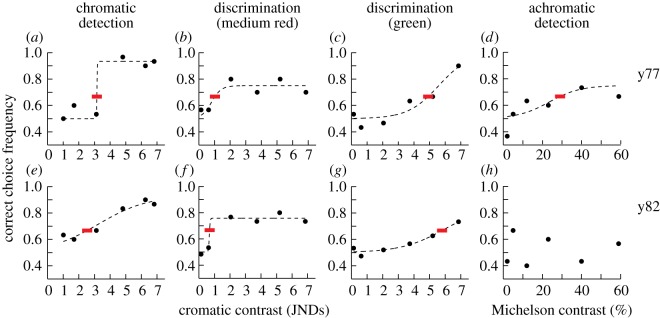
Psychometric functions (dashed lines) fitted to behavioural data from two zebra finches, y77 (*a–d*) and y82 (*e–h*). Filled circles represent the correct choice frequencies for 30 trials and red bars indicate thresholds at 66.7% (see Material and methods). Figures with psychometric data from all birds and at all conditions can be found in the electronic supplementary material.

In the tests of chromatic detection, only one disc was displayed and treated as the rewarded stimulus during each trial. One bird, w51, was removed from the experiments because of stress symptoms. Another bird, w60, was poorly motivated at this stage of testing, and kept only as company for b35 during the remaining trials. Among the three remaining birds, y77 and y82 immediately completed training during the first 20 trials, while b35 was ready for the tests after roughly 100 trials. In the tests, all three birds performed excellently at trials with high contrast stimuli, with correct choice frequencies about 90% (figure 5*a,e*). Detection thresholds are between 2.5 and 3.2 JNDs, thus higher than the lowest discrimination thresholds by a factor of approximately 2.5–3 (figure 4).

### 3.4. Achromatic detection

In the last series, without any further training, I tested the birds (b35, y77, y82) for the detection of grey discs of different intensity displayed on the same grey background used in the chromatic detection experiments. The birds were all confused by this test (figure 5*h*; electronic supplementary material) and only one bird produced correct choice frequencies above random level, and with a high detection threshold at 29% Michelson contrast (figure 5*d*).

## 4. Discussion

The results in this study show that in zebra finches, like humans [13], chromatic discrimination thresholds depend on the chromatic contrast between stimuli and the background. Another shared characteristic is that thresholds are higher for the detection, compared with the discrimination of chromatic contrast. The details of these findings, and their importance for the ecology and evolution of bird colour vision are discussed below.

### 4.1. Discrimination thresholds and the ‘dipper function’

I conclude that colour discrimination thresholds increase when stimuli have a stronger contrast to the background (figure 4). For two of the birds (w51, b35), this relationship is confounded by high discrimination thresholds for the yellow background (figure 4). Despite their successful training, and the instruction trials given at the beginning of each session, these two birds may have been confused by the background shifts. Alternatively, the thresholds of these two birds truly are higher for the yellow compared with the grey background, although there are no obvious explanations for such a difference. The yellow background was included in the tests to examine if there is a different between a shift in receptor adaptation colinear to the variation in saturation of the stimulus discs (green, grey, medium red backgrounds), and a shift in hue orthogonal to saturation (yellow background). Unfortunately, this issue cannot be resolved with the large spread of behavioural responses for the yellow background (figure 4).

In agreement with the RNL model and a Weber fraction of 0.1 in the LWS-cone mechanisms (equation (2.6)), the lowest discrimination threshold is very close to 1 JND (figures 4 and 5*f*). This threshold was not found in the detection task, carried out at the background chromaticity, but for the discrimination between stimuli with modest contrast against the background (figures 4 and 5*a,b,e,f*). This causes a ‘dip’ in the function that describes thresholds as a function of background contrast.

The ‘dipper function’ is well described in human colour vision, where it appears in the use of stimulus ‘pedestals’ [28,29]. A pedestal is an achromatic or chromatic contrast against the background that is shared by, and usually has the same spatial and temporal properties, as the stimuli. For example, the task may be to discriminate between two colour stimuli of the same brightness, where one of them has the same chromaticity as the background and therefore cannot be distinguished. In this case, the task is a matter of detecting a chromaticity shift from the background. However, if the stimuli retain their chromaticity but are placed on luminance pedestals, meaning that they are given a shared achromatic contrast against the background, they are both distinct and the detection task becomes a discrimination task.

Several studies on humans have shown that thresholds first decrease as pedestal contrast is raised slightly above zero and then increase as the pedestal grows even stronger, hence the threshold ‘dip’. The dipper function is not specific to colour vision, but occurs also in other visual modalities such as spatial contrast discrimination [30]. Why pedestals facilitate contrast detection is still not clear but it has been suggested that the demarcation of the stimuli aid retinal integration or neuronal gain mechanisms [29,31].

For the zebra finches, the pedestals were composed of a stable achromatic contrast together with a variable chromatic contrast. Just as for human, thresholds decrease with a modest level of pedestal contrast, but increases again when the chromatic contrast of the pedestals continues to increase (figure 4). In one case, background intensity was raised to test the effects of a decrease in achromatic pedestal contrast, but this did not affect discrimination thresholds (figure 3). Again, this is parallel to human colour vision where any small amount of pedestal contrast facilitates discrimination, but where stronger pedestal contrast has little effect unless it has the same modality (achromatic or chromatic) as the stimulus variation. This is very useful for the design of colour vision tests because chromatic stimuli can be placed upon achromatic pedestals to disfavour the use of achromatic cues.

Besides similar spatial tuning of colour vision [19], the correlation between discrimination thresholds and background contrast and the presence of the dipper function in zebra finches are additional examples of shared characteristics of colour vision in mammals and birds despite their fundamentally different physiology.

### 4.2. Colour learning

To investigate discrimination thresholds at zero chromatic contrast against the background, I trained birds for the discrimination between the stimuli on achromatic pedestals displayed on a background with the same chromaticity as the rewarded stimulus (figure 2). This training failed completely in all birds (electronic supplementary material, S3).

The achromatic (Michelson) contrast between the rewarded stimulus and the red background was 25%, which is well above roughly 10% brightness discrimination threshold determined in other birds (see below). However, brightness vision has not been tested in zebra finches, and the achromatic sensitivity may be very low for very large objects [19]. If the zebra finches failed to detect the achromatic pedestals, discrimination would be made between non-rewarded discs, and a rewarded alternative with no contrast separation from the background so that the task becomes the odd and probably confusing challenge of choosing sides without discs to get the food rewards.

Another possibility is that a complete adaptation to the background would shift the colour of the rewarded stimulus into the neutral point of the chromaticity diagram. Consequently, the task of discriminating discs in an LWS-dominated region would become a matter of discriminating discs in an MWS-dominated region of colour space, where the rewarded alternative is the most unsaturated alternative (figure 2). It is worth noting that birds did not simply choose the most saturated colours. For this background, this would give a preference for all but the rewarded discs and thus, very few correct choices. However, the correct choice frequencies were not abnormally low, but at random level even at the very first choices (electronic supplementary material, S3). Instead, it is more likely that the shift confused the zebra finches because they learn colours by some categorical identity. Although categorical concepts, like ‘red’ and ‘green’, usually are restricted to human perception, it has been shown that domestic chicks generalize colours within categorical boundaries [32]. This study was not designed to investigate colour learning, but the method of shifting colour identity by background adaptation and test for colour preference may prove a useful technique to further explore generalization and categorical boundaries in animal colour vision.

### 4.3. Achromatic contrast detection

The last experiment shows that birds did not generalize between chromatic and achromatic detection (figure 5). For achromatic detection, one of three birds produced choice frequencies above the random level (figure 5*d*), and only with poor performance that resulted in a very high threshold of 29% Michelson contrast. This threshold is substantially higher than the threshold for brightness discrimination in other bird species: 9% in budgerigars [33] and 13% in pigeons [34]. The zebra finches were not only confused by the transition between the chromatic and the achromatic task, but there were no signs of learning achromatic detection (i.e. performance did not improve in late trials; electronic supplementary material, S8). It has been claimed that for large static stimuli displayed on a homogeneous background, detection is mainly driven by chromatic vision [9,11,19]. The results are in line with this idea, and further suggest that in zebra finches, a low sensitivity for achromatic contrast in large-field stimuli is coupled with a slow or absent learning of such cues.

### 4.4. Colour thresholds and visual ecology

The understanding of receiver perception is central to studies of the ecology and the evolution of sensory signals [35]. The results on colour discrimination in zebra finches help us understand and model colour vision and colour trait variation in birds.

First, the dipper function, the difference in thresholds between detection and discrimination, demonstrates that the specific behaviour investigated is crucial for making accurate model predictions of colour vision performance. Interestingly, thresholds are about two to three times higher for detection compared with discrimination in both zebra finches and humans [28,29]. Hopefully, future investigations will clarify if a difference of this magnitude is a general characteristic of colour vision in birds and mammals.

Second, discrimination thresholds depend on the colour of the background. This effect is not accounted for in the popular RNL model [9]. For the red beaks of male zebra finches, colour variation close to the threshold given by the RNL model (1 JND) is meaningful only for a red background, and not for the more natural backgrounds of brown and green vegetation.

**Figure 6.**
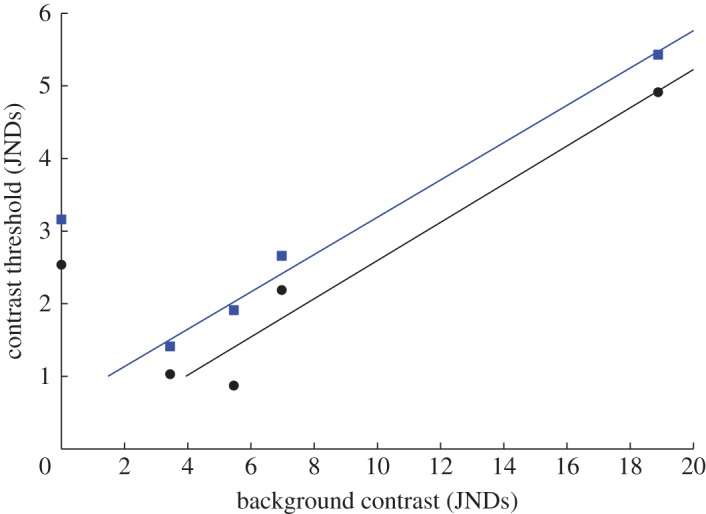
Linear functions fitted to data on colour discrimination at modest to strong background contrast in two zebra finches (Y77, blue squares and y82, black circles). Contrast threshold varies significantly with background contrast (Pearson's correlation: Y77, *p* = 0.001; y82, *p* = 0.039). The functions have slopes of 0.257 and 0.263, *R*^2^-values of 0.99 and 0.96, and intersect with the 1 JND threshold at 1.48 and 3.94 JNDs background contrast in Y77 and Y82, respectively. The data on detection at zero background contrast are included to show the dipper function, the dip in thresholds at low to medium background contrast. Background contrast is calculated as in figure 4.

Fortunately, it is possible to quantify these effects in order to calibrate the models. In the two best-performing birds (y77 and y82), the lowest discrimination thresholds are close to the limit predicted from the RNL model with a noise level of 0.1 in the LWS-cone mechanism. The increase in threshold at stronger background contrast is well described by linear functions with practically identical slopes in both birds (figure 6). From this, we can make predictions. For example, the contrast between the red beak of a zebra finch and green vegetation is roughly 9.5 JNDs. At this level of background contrast, the discrimination threshold is about 2.5 JNDS. The blue crest of a blue tit has a contrast of about 12.5 JNDs against green vegetation, and thus a meaningful variation above 3.2 JNDs. This correction for background contrast could easily be incorporated into the RNL model.

An exciting prediction that follows from these results is that the variation in colour traits should be larger for traits that are more strongly contrasting the background. Small variation in highly conspicuous traits is probably not detected by the observer unless the colour traits are displayed in a position with a favourable background. Furthermore, this may form a constraint in the evolution of highly saturated colour traits and help to explain the global distribution of bird colours within the bird colour space [36].

This investigation adds knowledge to a rapidly growing body of research on how visual performance in birds relates to receptor adaptation, including studies on colour vision in dim light [12], spectral sensitivity [10,11,37] and colour constancy [38]. So far, we are only scratching the surface on this field of science. Hopefully, more general ideas will soon be developed that allow for unifying descriptions of colour perception and the evolution of colour traits in the same theoretical framework.

## Supplementary Material

Supplementary methods - photographs of the experimental setup.

## Supplementary Material

Supplementary results- additional figures showing all behavioural training and test results.

## Supplementary Material

Supplementary data - data on colour spectra and the sensitivity of zebra finch cones. Supplementary movies of the experiments

## Data accessibility

All relevant data are within the article and its electronic supplementary material.
