# Supplementary material for: Colour vision and background adaptation in a passerine bird, the zebra finch (Taeniopygia guttata)
Source: R Soc Open Sci. 2016 Sep 14;3(9):160383. doi: 10.1098/rsos.160383 (PMC5043321; doi:10.1098/rsos.160383)
Supplement: Supplementary methods - photographs of the experimental setup. [file rsos160383supp1.pdf]

Supplementary Methods

Colour vision and background adaptation in a passerine bird, the zebra finch (*Taeniopygia guttata*)

Olle Lind

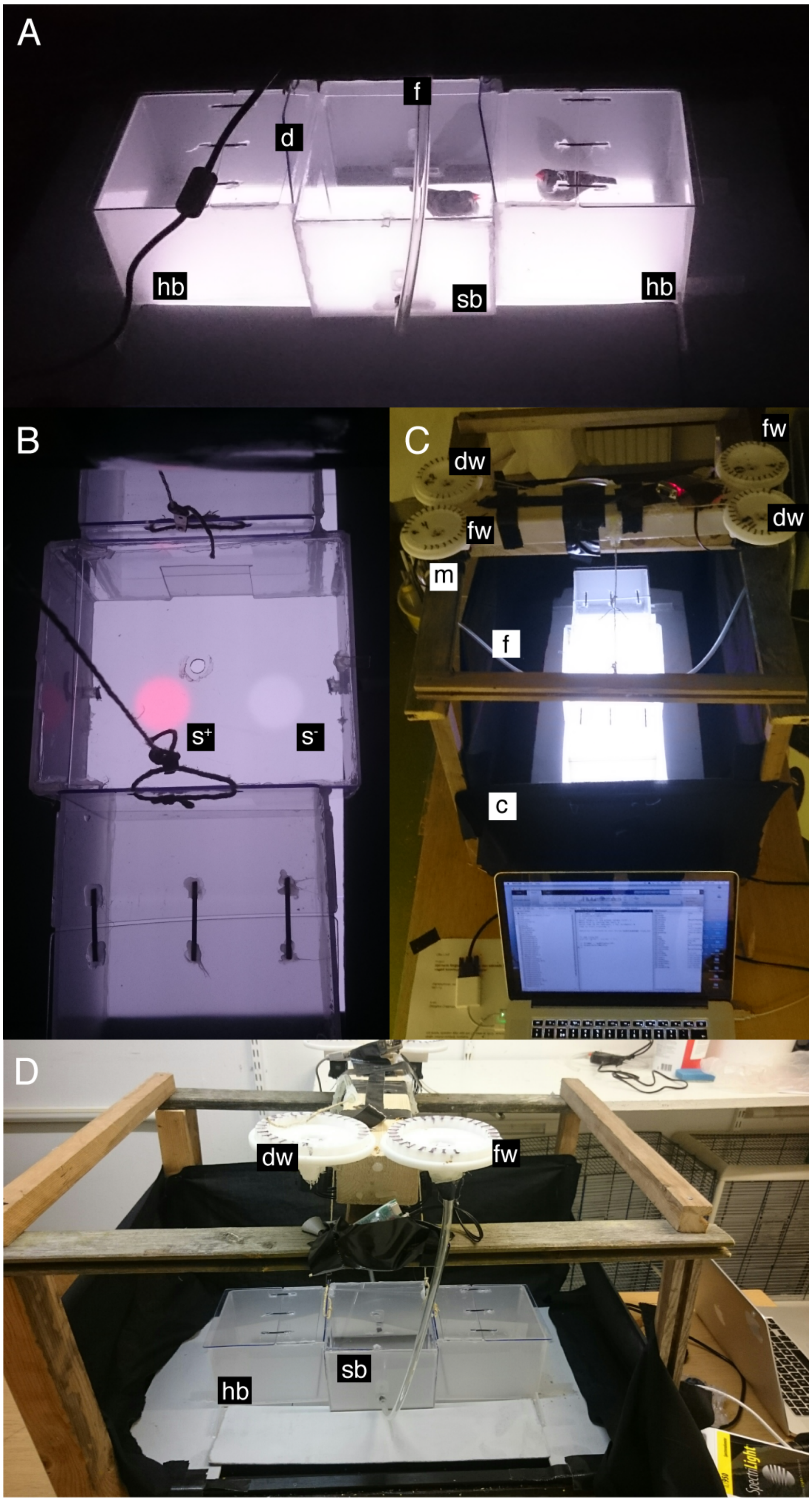

Figure 1. The test apparatus view from the side (A,D), top (B), and front (C). Central components are identified; c - black curtain, d – clear glass door, dw –wheel to withdraw door, f - feeder tube, fw – feeder wheel, hb – house-box, m – wheel-motor, S^+ - reinforced stimulus, S^- - negative stimulus, sb – stimulus box,. Note in (C) and (D), the room lighting is on for photography. During tests, all room lighting was turned off.
