# Supplementary material for: Colour vision and background adaptation in a passerine bird, the zebra finch (Taeniopygia guttata)
Source: R Soc Open Sci. 2016 Sep 14;3(9):160383. doi: 10.1098/rsos.160383 (PMC5043321; doi:10.1098/rsos.160383)
Supplement: Supplementary results- additional figures showing all behavioural training and test results. [file rsos160383supp2.pdf]

Supplementary Results
Colour vision and background adaptation in a passerine bird, the zebra finch (*Taeniopygia guttata*)
Olle Lind

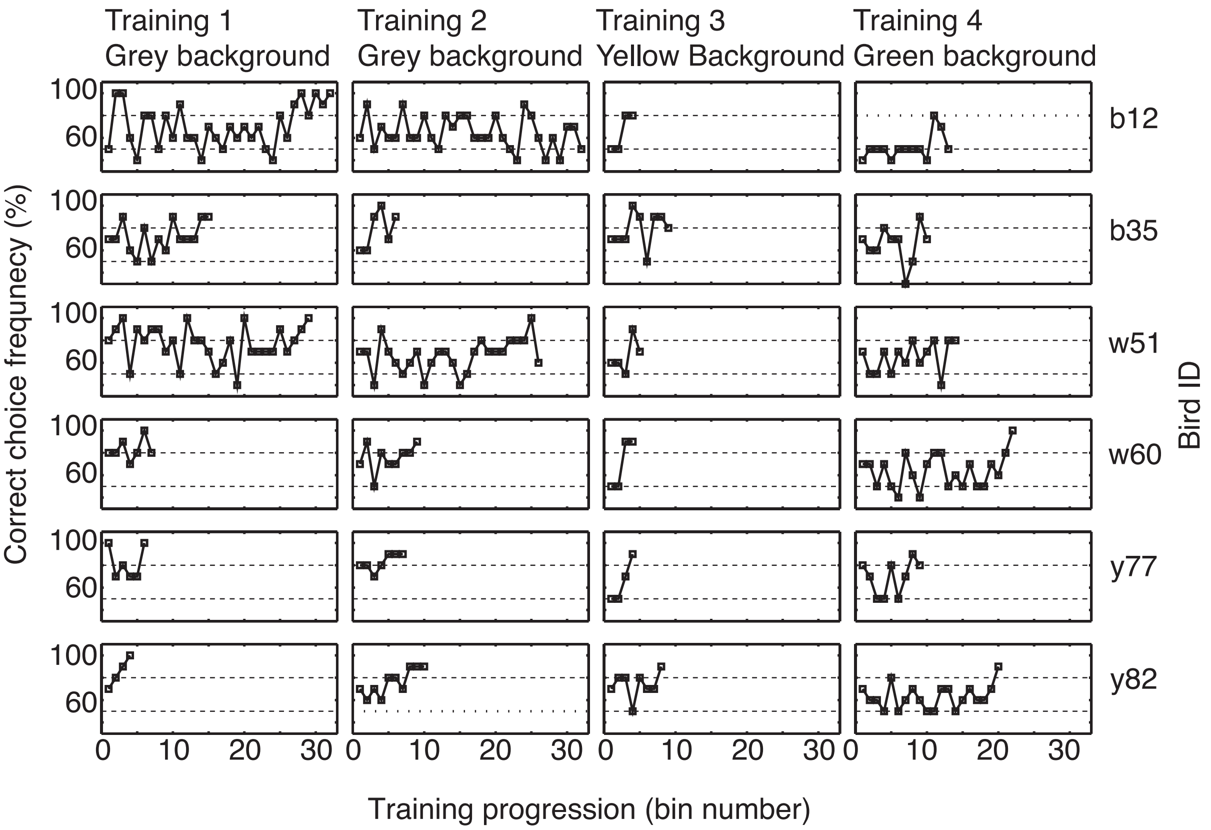

Figure S2. Training results for the discrimination series 1. Each square represents the correct choice frequency for 10 trials and dashed lines indicate 50% and 80% correct choices. Initial trials are trimmed to make bin sizes even. In training 1 and 2, birds discriminated between discs of strong and medium colour contrast respectively and in training 3 and 4, birds discriminated between a mixture of these disc contrasts. Training was considered successful for at least 80% correct choices during 40 consecutive trials in training 1 and 2 and during 20 consecutive trials in training 3 and 4 (see main text for details).

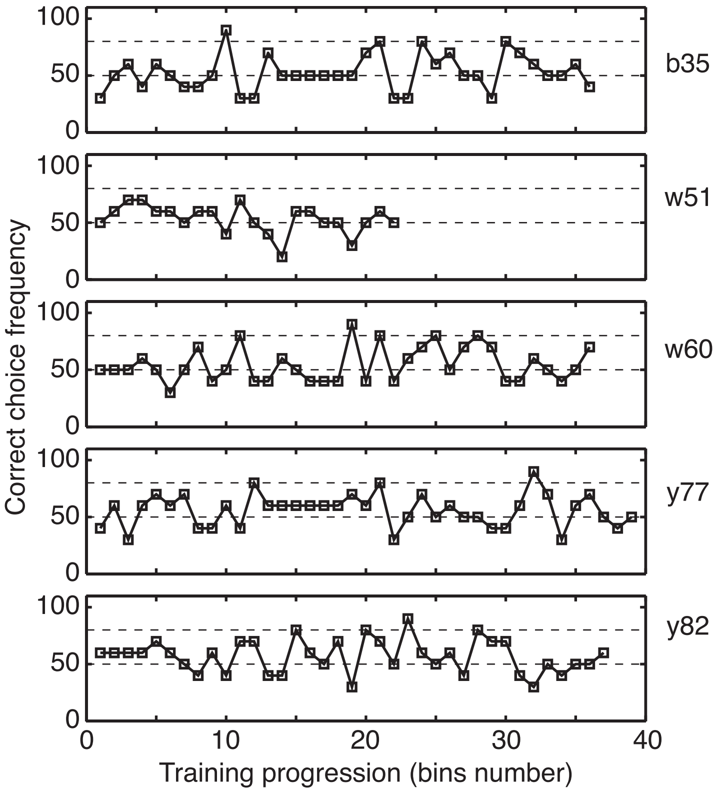

Figure S3. Training results for discrimination of discs on a red background with the same colour as the colour of the rewarded stimulus. Notations are as in figure S2.

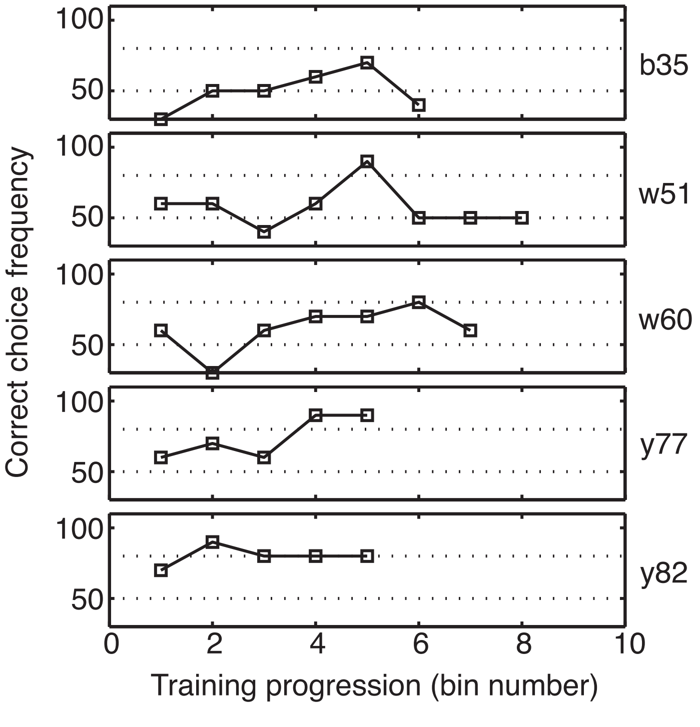

Figure S4. Training results for a medium red background. Notations as in figure S2. Training was considered successful for at least 80% correct choices during 20 consecutive trials

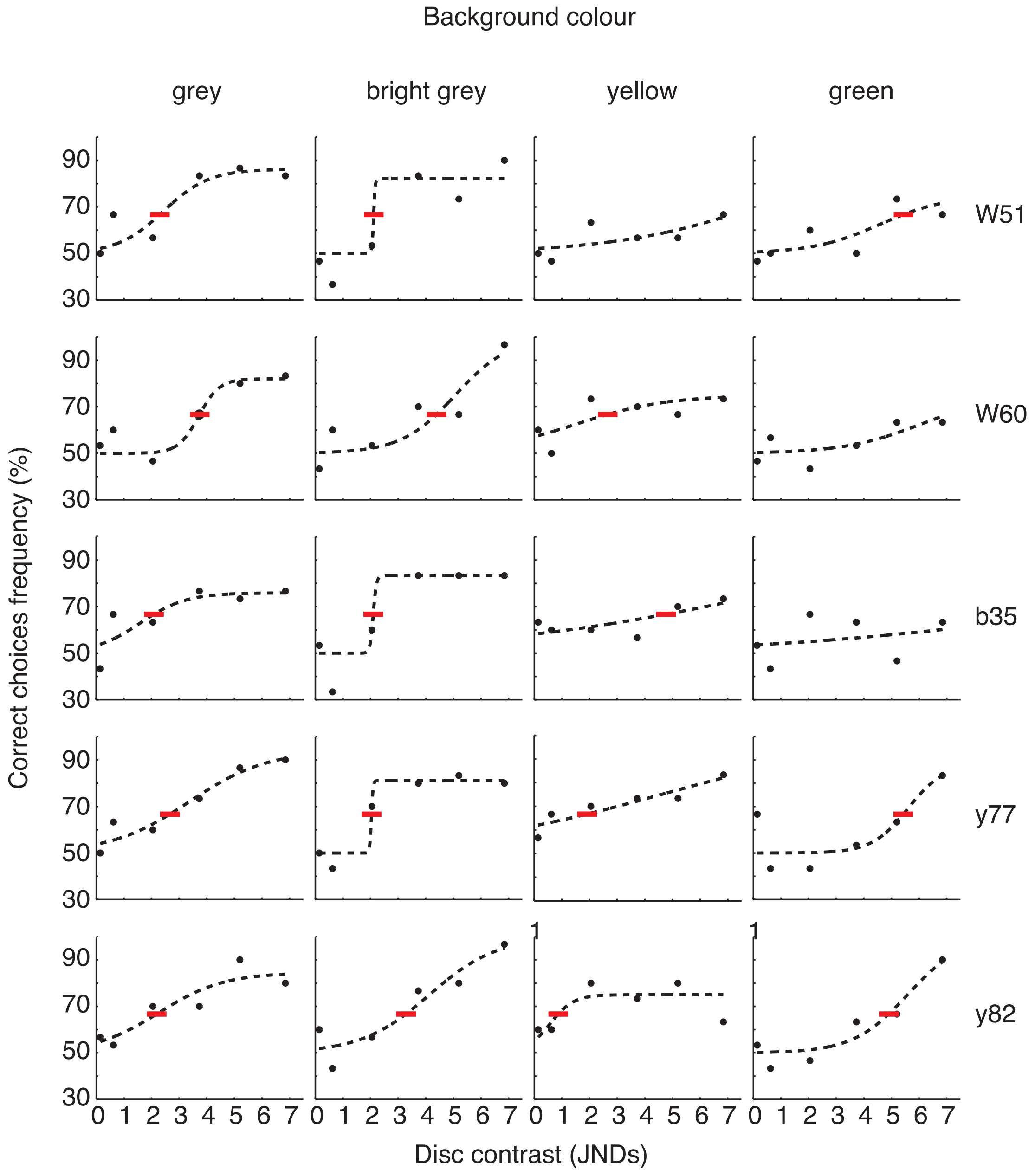

Figure S5. Logistic psychometric functions fitted to the behavioural data for five zebra finches. Circles show the correct choice frequencies for 30 trials at each of six contrast levels, and the red bars indicate the interpolated threshold at 66.7% correct choices.

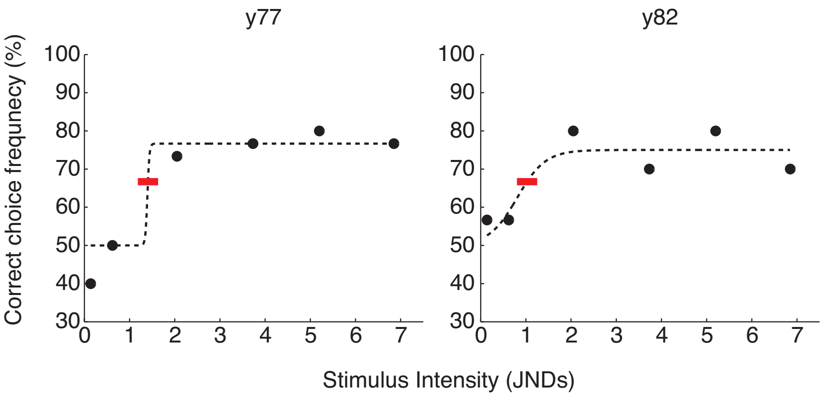

Figure S6. Behavioural data and psychometric functions for discrimination between discs on a medium red background. Notations as in figure S5.

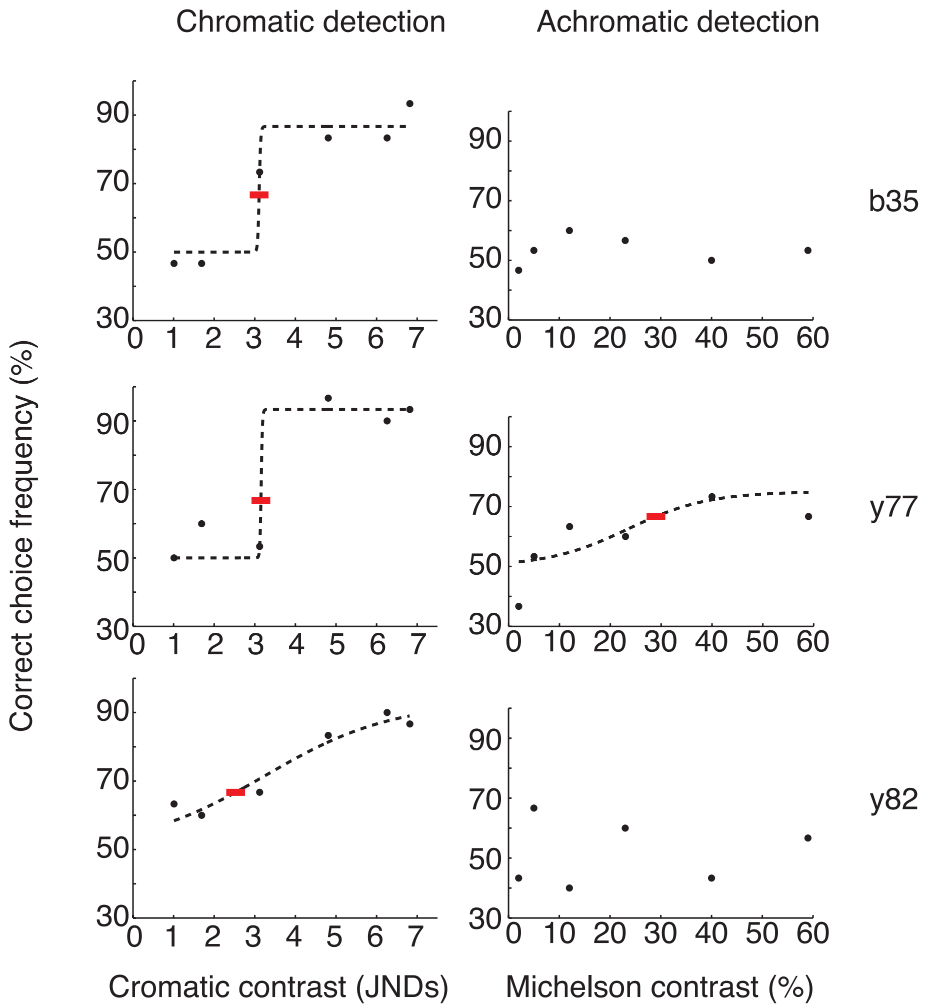

Figure S7. Behavioural data and psychometric functions for the detection of discs with chromatic or achromatic contrast on a bright grey background. Notations as in figure S5.

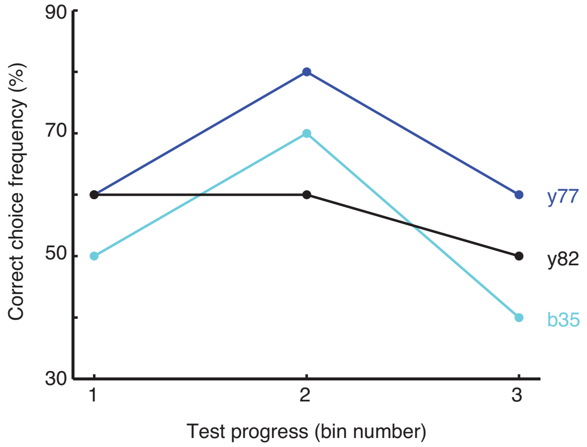

Figure S8. Tests of achromatic detection of grey discs on a bright grey background. The correct choice frequencies are for the strongest achromatic contrast during the first, middle, and latest 10 trials.
